# Boiling histotripsy exhibits anti-fibrotic effects in animal models of liver fibrosis

**DOI:** 10.1038/s41598-024-66078-x

**Published:** 2024-07-02

**Authors:** Chanmin Joung, Jeongmin Heo, Ki Joo Pahk, Kisoo Pahk

**Affiliations:** 1 Graduate School of Biomedical Sciences, University of Texas Southwestern Medical Center, Dallas, TX 75235 USA; 2 Center for Bionics, Biomedical Research Institute, Korea Institute of Science and Technology (KIST), Seoul, 02792 Republic of Korea; 3 Department of Biomedical Engineering, Kyung Hee University, 1732, Deogyeong-daero, Giheung-gu, Yongin-si, Gyeonggi-do 17104 Republic of Korea; 4 Department of Nuclear Medicine, Korea University College of Medicine, 73, Inchon-ro, Seongbuk-gu, Seoul, 02841 Republic of Korea

**Keywords:** High intensity focused ultrasound, Boiling histotripsy, Acoustic cavitation, Liver fibrosis, Anti-fibrotic treatment, Liver regeneration, Biomedical engineering, Liver cirrhosis, Liver fibrosis

## Abstract

Liver fibrosis is a hallmark of chronic liver disease which could lead to liver cirrhosis or liver cancer. However, there is currently lack of a direct treatment for liver fibrosis. Boiling histotripsy (BH) is an emerging non-invasive high-intensity focused ultrasound technique that can be employed to mechanically destruct solid tumour at the focus via acoustic cavitation without significant adverse effect on surrounding tissue. Here, we investigated whether BH can mechanically fractionate liver fibrotic tissue thereby exhibiting an anti-fibrotic effect in an animal model of liver fibrosis. BH-treated penumbra and its identical lobe showed reduced liver fibrosis, accompanied by increased hepatocyte specific marker expression, compared to the BH-untreated lobe. Furthermore, BH treatment improved serological liver function markers without notable adverse effects. The ability of BH to reduce fibrosis and promote liver regeneration in liver fibrotic tissue suggests that BH could potentially be an effective and reliable therapeutic approach against liver fibrosis.

## Introduction

Liver fibrosis is an excessive wound-healing process in chronic liver diseases, including alcoholic liver disease, non-alcoholic fatty liver disease, infections caused by hepatitis B or C viruses, as well as genetic and autoimmune diseases^1^. The clinical manifestation of liver fibrosis is characterised by excessive accumulation of extracellular matrix (ECM), thereby destroying normal liver architecture and function^2^. Unmanaged liver fibrosis may unavoidably lead to cirrhosis, which is a key risk factor for liver failure and liver cancer, and which affects 1% to 2% of global population, resulting in over 1 million deaths every year^3,4^. However, currently, there is no approved therapy for the treatment of liver fibrosis and cirrhosis^5^.

Boiling histotripsy (BH) is a well-established novel high intensity focused ultrasound (HIFU) technique which uses a number of short HIFU pulses (of the order of milliseconds) with very high peak positive (*P*_+_ > 40 MPa) and peak negative (*P*_−_ < 10–15 MPa) pressures to mechanically destroy target soft tissue at HIFU focus via acoustic cavitation without inducing thermal damage^6–8^. In contrast to traditional HIFU thermal ablation, BH can induce a more distinct boundary between fractionated and unfractionated region because BH is not significantly affected by heat sink effect. Studies have shown that BH fractionates various soft tissue types, including the heart, kidney, and liver, as well as cancer cells such as MDA-MB-231 breast cancer, renal cell carcinoma, and B16 melanoma, whilst ensuring no thermal injury to the surrounding BH lesion^7–15^. Furthermore, it has been recently reported that BH can also promote the physiologic wound healing processes and tissue regeneration whilst preventing hepatic fibrosis and the formation of permanent scar tissue in the BH-treated area of the normal liver in vivo^16^. In the present study, we therefore aimed to examine whether BH can mechanically fractionate fibrotic liver tissue, thereby demonstrating an anti-fibrotic effect in an animal model of liver fibrosis.

## Results

### BH attenuates thioacetamide (TAA)-induced liver fibrosis

**Figure 1:**
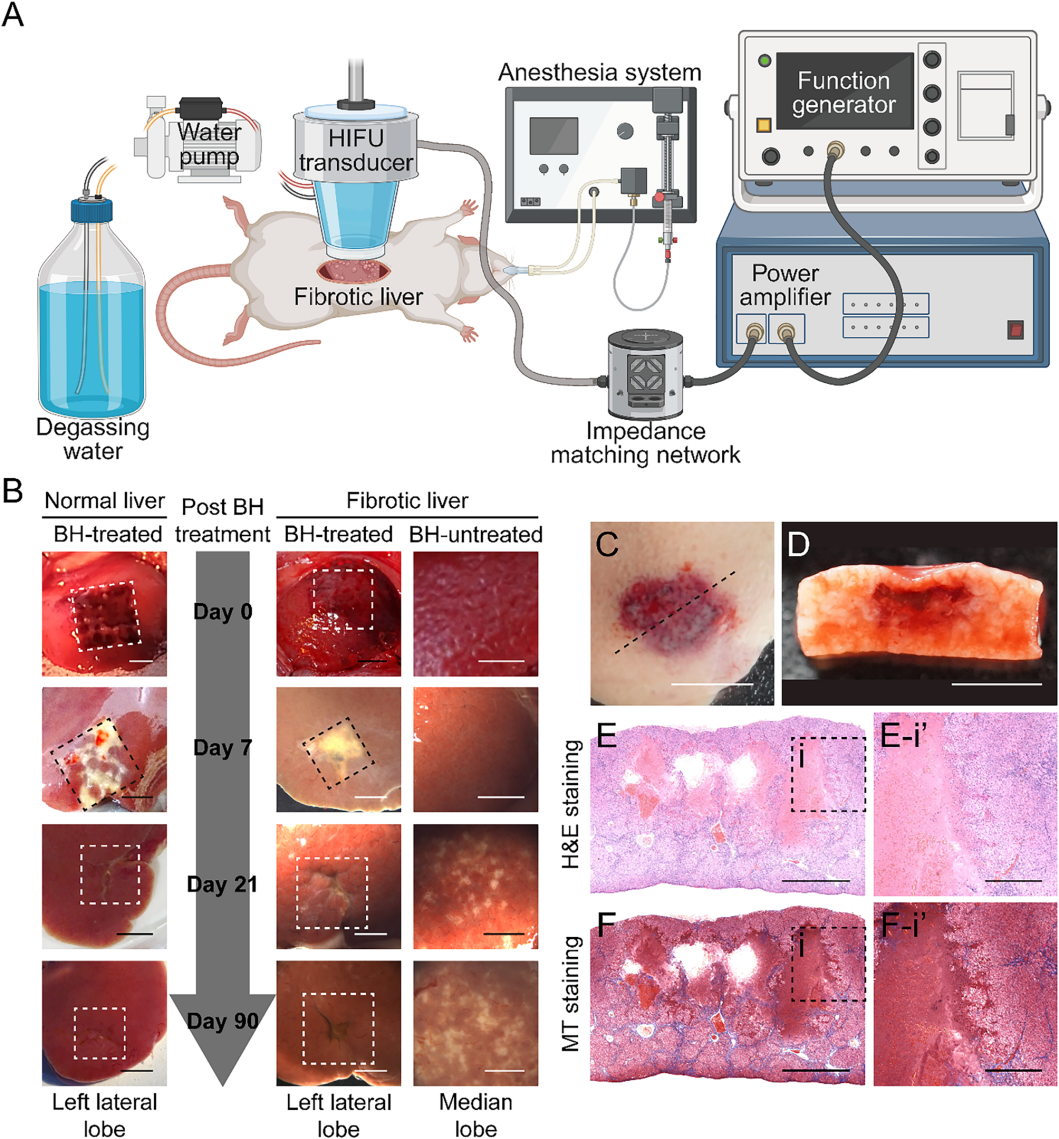
(**A**) A schematic diagram of the experimental setup for boiling histotripsy (BH) treatment. HIFU; high intensity focused ultrasound. (**B**) Chronological gross morphological changes of both normal and fibrotic liver tissue on days 0, 7, 21 and 90 after the BH treatment. The square with broken line indicates BH-treated regions. (Scale bar, 5 mm). (**C–D**) Gross and cross-sectional images of fibrotic liver tissues after BH treatment on Day 0. (**C**) Top view of BH-treated fibrotic liver tissue after cardiac perfusion. The broken line indicates a cross–section line for histological observation. (Scale bar, 10 mm). (**D**) Cross section of BH-treated fibrotic liver tissue after cardiac perfusion. (Scale bar, 10 mm). (**E–F**) Histological images of (**E**) haematoxylin and eosin (H&E) or (**F**) Masson’s trichrome–stained liver tissues collected on Day 0. (Scale bar, 1 mm). Images (i) show the highlighted areas (between BH-treated and untreated areas) in (**E** to **F**, enclosed by a square with broken lines) at higher magnifications. (Scale bar, 250 µm; magnification ×100).

**Figure 2:**
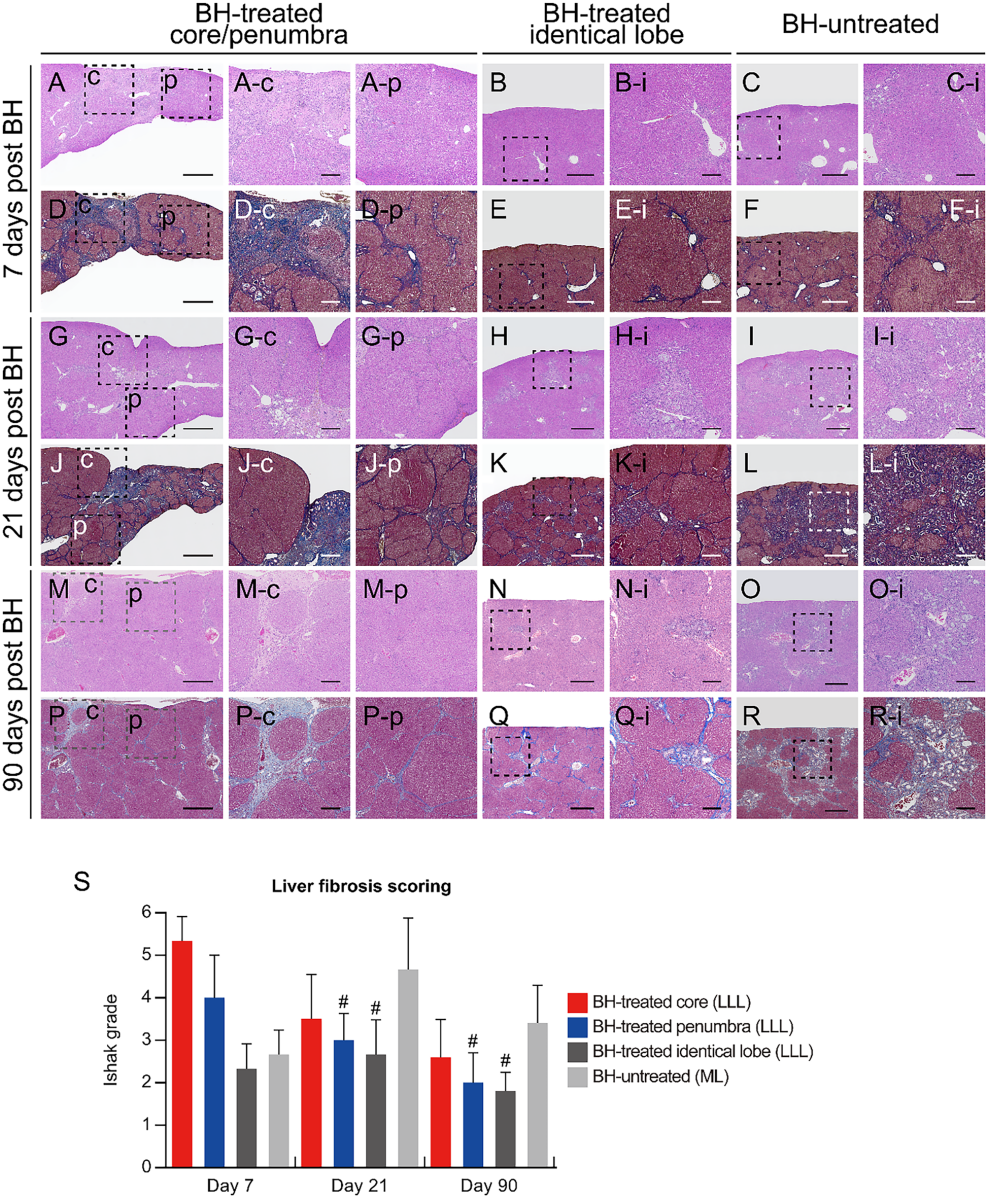
Cross sectional images of the fibrotic liver tissues after BH treatment. (**A–R**) Histological images of (**A–C, G–I, M–O**) haematoxylin and eosin (H&E) or (**D–F, J–L, P–R**) Masson’s trichrome stained liver tissues collected on days 7, 21, and 90 after the BH treatment. (**A, D, G, J, M, P**) BH-treated region in the left lateral lobe (LLL). (**B, E, H, K, N, Q**) The BH-unaffected region in the BH-treated LLL. (**C, F, I, L, O, R**) BH–untreated median lobe (ML). (Scale bar, 1 mm). Images (c), (p), and (i) show the highlighted areas in (**A** to **R**, square with broken lines) at higher magnifications. (Scale bar, 250 µm; magnification ×100). c: BH-treated core, p: BH-treated penumbra. (**S**) Liver fibrosis index score for each region of fibrotic liver samples at days 7, 21, and 90 after the BH treatment. n = 6 for sham, n = 6 for Day 7, n = 5 for Day 21, n = 3 for Day 90. All values are shown as means ± standard deviation (SD, ^#^*P* < 0.05 vs. BH-untreated (ML) of each time point).

A fibrotic liver is generally stiffer than a healthy normal liver. In the present study, BH treatment was successfully performed to mechanically fractionate fibrotic liver tissue in vivo (Fig. 1A to F) with the BH protocol previously used for normal liver fractionation^16^. The morphological chronological changes of the liver after the BH treatment are shown in Fig. 1B. In the acute phase, 7 days after the BH treatment, both the BH-treated core and penumbra showed relatively higher liver fibrosis scores than the BH-untreated lobe, although the difference was not statistically significant. There was also no significant difference between the BH-treated identical lobe and the untreated lobe (Fig. 2A to F, and S). In contrast, 21 and 90 days after the BH treatment, both the BH-treated penumbra and BH-treated identical lobe showed significantly reduced liver fibrosis scores compared to the BH-untreated lobe. Furthermore, the BH-treated core also exhibited a lower fibrosis score than the BH-untreated lobe (Fig. 2G to R, and S).

**Figure 3:**
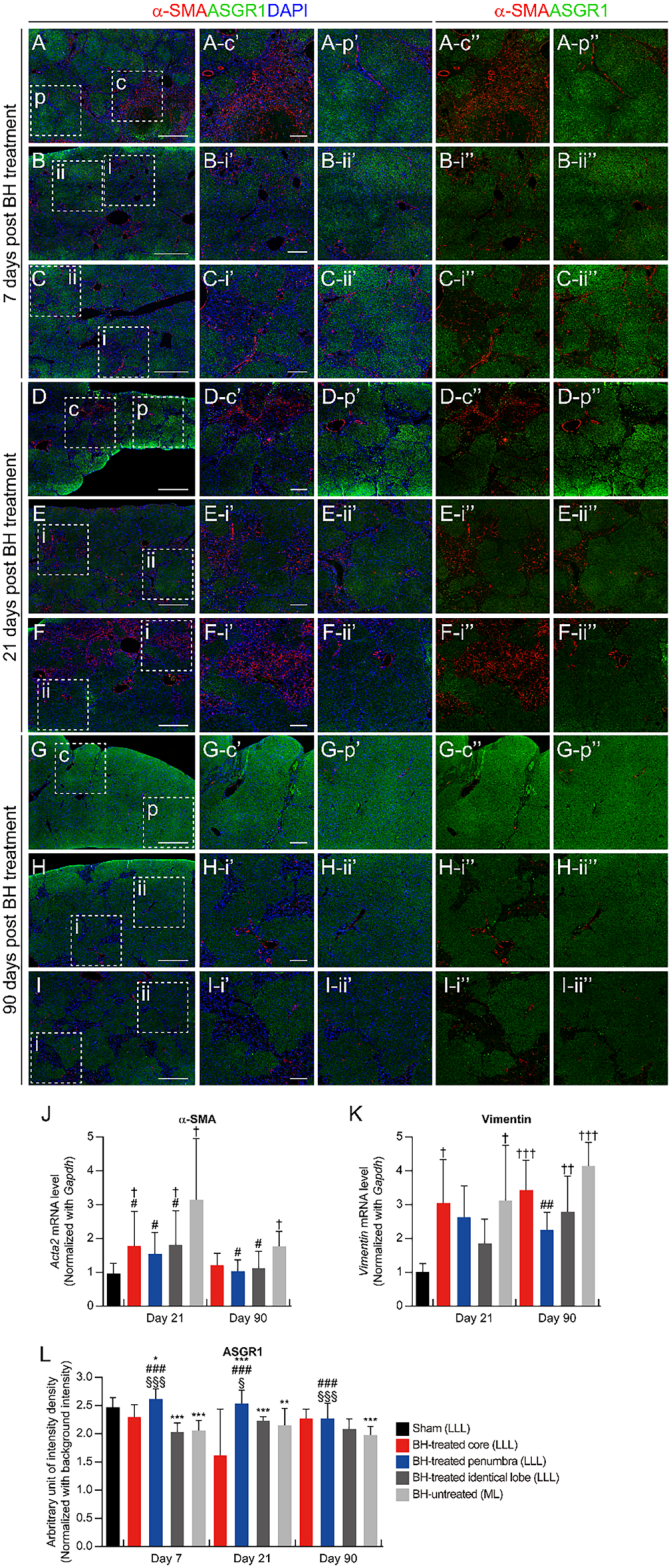
Clearance of myelofibroblasts and replenishment of lost hepatocytes after BH treatment. (**A–I**) Representative immunohistochemistry images of myelofibroblasts (α–SMA) and hepatocytes (ASGR1) in the cross–sectioned liver tissues collected on days 7, 21, and 90 after the BH. (**A, D, G**) BH-treated region in the LLL. (**B, E, H**) The BH-unaffected region in the BH-treated LLL. (**C, F, I**) BH-untreated ML. (Scale bar, 1 mm). Images (c′), (p′), (i′), and (ii′) show the highlighted areas in (A to I, square with broken lines) at higher magnifications with three channels (Red, Green, and Blue). Images (c″), (p″), (i″), and (ii″) show the highlighted areas in (A to I, square with broken lines) at higher magnifications with two channels (Red and Green). (Scale bar, 250 µm; magnification ×100). c: BH-treated core, p: BH-treated penumbra, i: severe fibrotic region, ii: mild fibrotic region. (**J**) Transcriptome levels of α-SMA for each region of the fibrotic liver at days 21 and 90 after the BH treatment. n = 6 for sham, n = 6 for Day 21, n = 4 for Day 90. (**K**) Transcriptome levels of Vimentin for each region of the fibrotic liver at days 21 and 90 after the BH treatment. n = 5 for sham, n = 8 for Day 21, n = 5 for Day 90. (**L**) Quantification of the fluorescence intensity of ASGR1 in each region of fibrotic liver samples at days 7, 21, and 90 after the BH treatment. n = 20 for sham, Day 7: n = 8 for BH-treated core (LLL), n = 14 for BH-treated penumbra (LLL), n = 26 for BH-treated identical lobe (LLL), n = 34 for BH-untreated (ML), Day 21: n = 4 for BH–treated core (LLL), n = 22 for BH-treated penumbra (LLL), n = 20 for BH-treated identical lobe (LLL), n = 37 for BH-untreated (ML), Day 90: n = 9 for BH-treated core (LLL), n = 26 for BH-treated penumbra (LLL), n = 47 for BH-treated identical lobe (LLL), n = 40 for BH-untreated (ML). All values are shown as means ± SD (^†^*P* < 0.05 vs. Sham (LLL), ^††^*P* < 0.01 vs. Sham (LLL), ^†††^*P* < 0.001 vs. Sham (LLL), *P < 0.05 vs. BH-treated core (LLL) of each time point, **P < 0.01 vs. BH-treated core (LLL) of each time point, ***P < 0.001 vs. BH–treated Core (LLL) of each time point, ^§^*P* < 0.05 vs. BH-treated identical lobe (LLL) of each time point, ^§§^*P* < 0.01 vs. BH-treated identical lobe (LLL) of each time point, ^§§§^*P* < 0.001 vs. BH-treated identical lobe (LLL) of each time point, ^#^*P* < 0.05 vs. BH-untreated (ML) of each time point, ^##^*P* < 0.01 vs. BH-untreated (ML) of each time point, ^###^*P* < 0.001 vs. BH-untreated (ML) of each time point).

Both α-smooth muscle-actin (α-SMA) and vimentin are well-known molecular markers for liver fibrosis^17,18^. On the 21st day after the BH treatment, all the BH-treated lobes showed reduced α-SMA expression compared to the BH-untreated lobe. 90 days after BH treatment, both the BH-treated penumbra and the BH-treated identical lobes exhibited reduced α-SMA expression compared to the BH-untreated lobe. Furthermore, the α-SMA expression of all the BH-treated lobes was not significantly different from the sham group. However, the BH-untreated lobe still showed a significant upregulation of α-SMA expression compared to the sham liver (Fig. 3A to I, and J). As shown in Fig. 3K, the expression of vimentin was upregulated in all TAA-induced fibrotic livers at 21 days after BH treatment, whereas the BH-treated penumbra exhibited reduced vimentin expression compared to the BH-untreated lobe at 90 days after the BH treatment.

**Figure 4:**
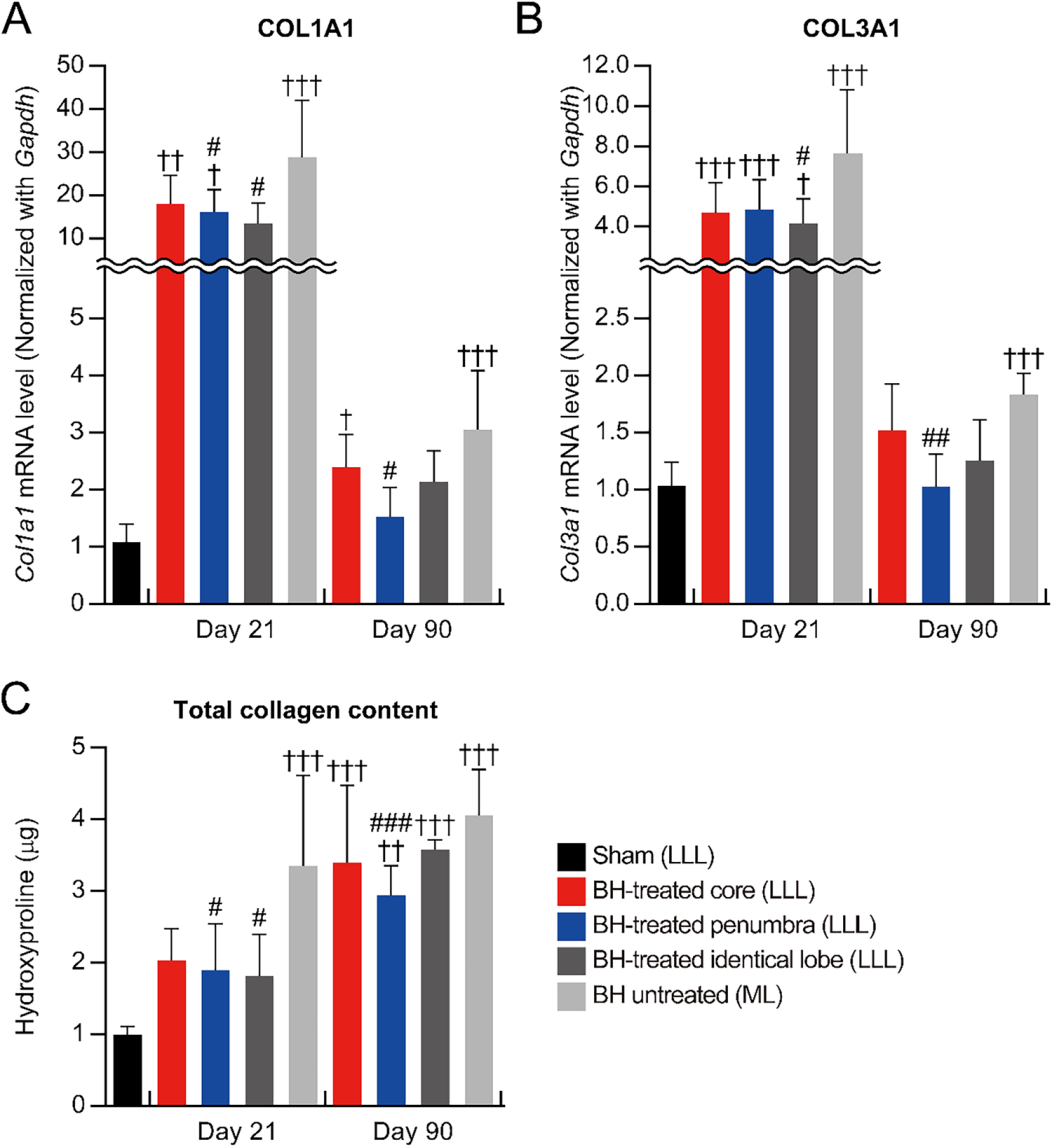
Collagen expression in fibrotic liver after BH treatment. (**A**) Transcriptome levels of collagen type I for each region of fibrotic liver at days 21, and 90 after the BH-treatment. n = 5 for sham, n = 6 for Day 21, n = 4 for Day 90. (**B**) Transcriptome levels of collagen type III for each region of fibrotic liver at days 21, and 90 after the BH-treatment. n = 6 for sham, n = 6 for Day 21, n = 4 for Day 90. (**C**) Quantification of the total collagen contents in each region of fibrotic liver at days 21 and 90 after the BH treatment. n = 6 for sham, n = 4 for Day 21, n = 4 for Day 90. All values are shown as means ± SD (^†^*P* < 0.05 vs. Sham (LLL), ^††^*P* < 0.01 vs. Sham (LLL), ^†††^*P* < 0.001 vs. Sham (LLL), ^#^*P* < 0.05 vs. BH-untreated (ML) of each time point, ^##^*P* < 0.01 vs. BH-untreated (ML) of each time point, ^###^*P* < 0.001 vs. BH-untreated (ML) of each time point).

Next, we evaluated the effect of BH on collagen content in TAA-induced liver fibrosis. After 21 days of the BH treatment, when compared with the BH-untreated lobe, the expression of collagen type I was reduced in both the BH-treated penumbra and the BH-treated identical lobe. Similarly, the expression of collagen type III was significantly decreased in the BH-treated penumbra. The total collagen content was also significantly decreased in the BH-treated penumbra and the BH-treated identical lobe (Fig. 4A to C). After 90 days of the BH treatment, the expression of collagen types I, III, and total collagen content was significantly reduced in the BH-treated penumbra compared to the BH-untreated lobe (Fig. 4A to C). These results indicate that BH treatment could reduce fibrotic tissues in a TAA-induced liver fibrosis animal model.

### BH promotes liver regeneration

**Figure 5:**
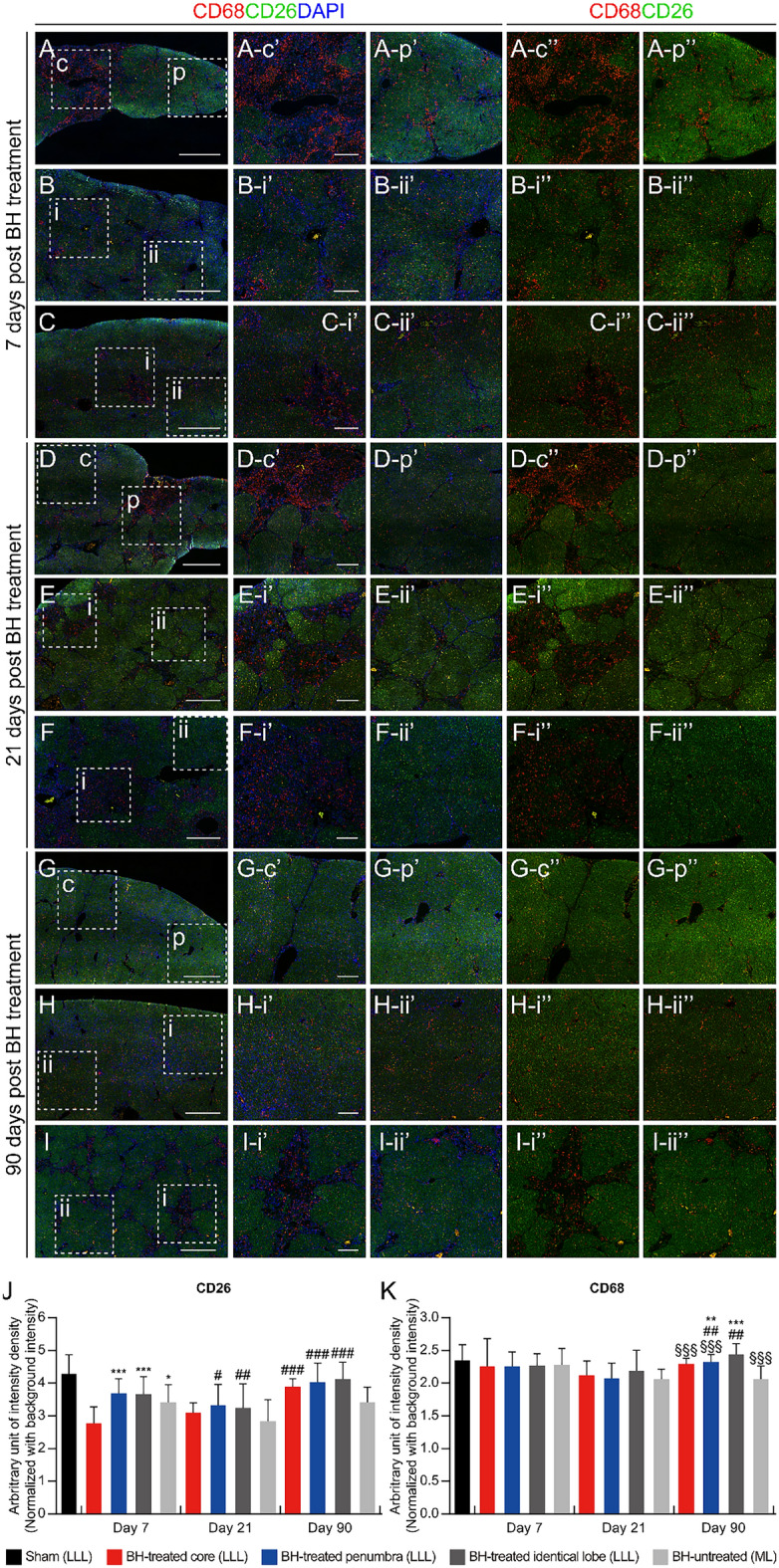
Replenishment of lost hepatocytes and inflammatory response after the BH treatment. (**A–I**) Representative immunohistochemistry images of hepatocytes (CD26) and CD68 positive cells in the cross–sectioned liver tissues collected on days 7, 21, and 90 after the BH. (**A, D, G**) BH-treated region in the LLL. (**B, E, H**) BH-unaffected region in the BH-treated LLL. (**C, F, I**) BH-untreated ML. (Scale bar, 1 mm). Images (c′), (p′), (i′), and (ii′) show the highlighted areas in (A to I, square with broken line) at higher magnifications with three channels (Red, Green, and Blue). Images (c″), (p″), (i″), and (ii″) show the highlighted areas in (A to I, square with broken line) at higher magnifications with two channels (Red and Green). (Scale bar, 250 µm; magnification ×100). c: BH-treated core, p: BH-treated penumbra, i: severe fibrotic region, ii: mild fibrotic region. (**J** and **K**) Quantification of the fluorescence intensity of CD26 and CD68 in each region of fibrotic liver samples at days 7, 21, and 90 after the BH treatment. n = 31 for sham, Day 7: n = 8 for BH-treated core (LLL), n = 19 for BH-treated penumbra (LLL), n = 55 for BH-treated identical lobe (LLL), n = 54 for BH-untreated (ML), Day 21: n = 5 for BH–treated core (LLL), n = 13 for BH-treated penumbra (LLL), n = 28 for BH-treated identical lobe (LLL), n = 25 for BH-untreated (ML), Day 90: n = 10 for BH-treated core (LLL), n = 21 for BH-treated penumbra (LLL), n = 54 for BH-treated identical lobe (LLL), n = 58 for BH-untreated (ML). All values are shown as means ± SD (*P < 0.05 vs. BH-treated core (LLL) of each time point, **P < 0.01 vs. BH-treated core (LLL) of each time point, ***P < 0.001 vs. BH-treated core (LLL) of each time point, ^§§§^*P* < 0.001 vs. BH-treated identical lobe (LLL) of each time point, ^#^*P* < 0.05 vs. BH-untreated (ML) of each time point, ^##^*P* < 0.01 vs. BH-untreated (ML) of each time point, ^###^*P* < 0.001 vs. BH-untreated (ML) of each time point).

The expression of asialoglycoprotein receptor 1 (ASGR1), a hepatocyte specific marker^19^, was significantly increased in the BH-treated penumbra compared to the BH-untreated lobe at all 7, 21, and 90 days after the BH treatment (Fig. 3A to I, and L). Next, we assessed the expression of CD26, which is also well-known as a hepatocyte marker^20,21^. On the 7th day after the BH treatment, both BH-treated and BH-untreated lobe showed significantly reduced CD26 expression compared to the sham group (Fig. 5A to C, and J). 21 days after the BH exposure, although both BH-treated and BH-untreated lobes still showed reduced CD26 expression compared to the sham, the BH-treated penumbra and BH-treated identical areas exhibited significantly increased CD26 expression compared to the BH-untreated lobe (Fig. 5D to F, and J). On the 90th day after the BH treatment, all BH-treated cores, BH-treated penumbras, and BH-treated identical lobes showed significantly increased CD26 expression compared to the BH-untreated lobe. Furthermore, the entire BH-treated lobe showed no statistically significant differences in CD26 expression compared to the sham, whereas the BH-untreated lobe still exhibited reduced CD26 expression compared to the sham group (Fig. 5G to I, and J). Taken together, these findings imply that treatment with BH promotes liver regeneration in an animal model of TAA-induced liver fibrosis.

### Effect of BH on liver inflammatory cells

We did not observe any ectopic infiltration of monocytes/macrophages one week after the BH treatment in the left lateral lobe (LLL) of the liver, compared with the basal CD68 expression level in non-fibrotic healthy liver tissue and in BH-untreated lobes with TAA-induced liver fibrosis (Fig. 5A to C, and K). On day 21 after the BH treatment, we observed a slight decrease in CD68 expression levels compared with the basal CD68 expression level, regardless of the BH-treated region (Fig. 5D to F, and K). At day 90, CD68 expression levels in the BH-treated lobe had recovered, and this recovery was more pronounced in distal regions compared to the core of BH treatment. In contrast, the decreased CD68 level in the BH-untreated lobe was not restored by day 90 (Fig. 5G to I, and K). At all 7, 21, and 90 days after the BH treatment, there were no statistically significant differences between the sham and the BH-treated or -untreated lobes. Thus, these results suggest that BH treatment on fibrotic liver tissue does not aggravate the inflammatory response.

### Effect of BH on body weight and serologic liver function markers

**Figure 6:**
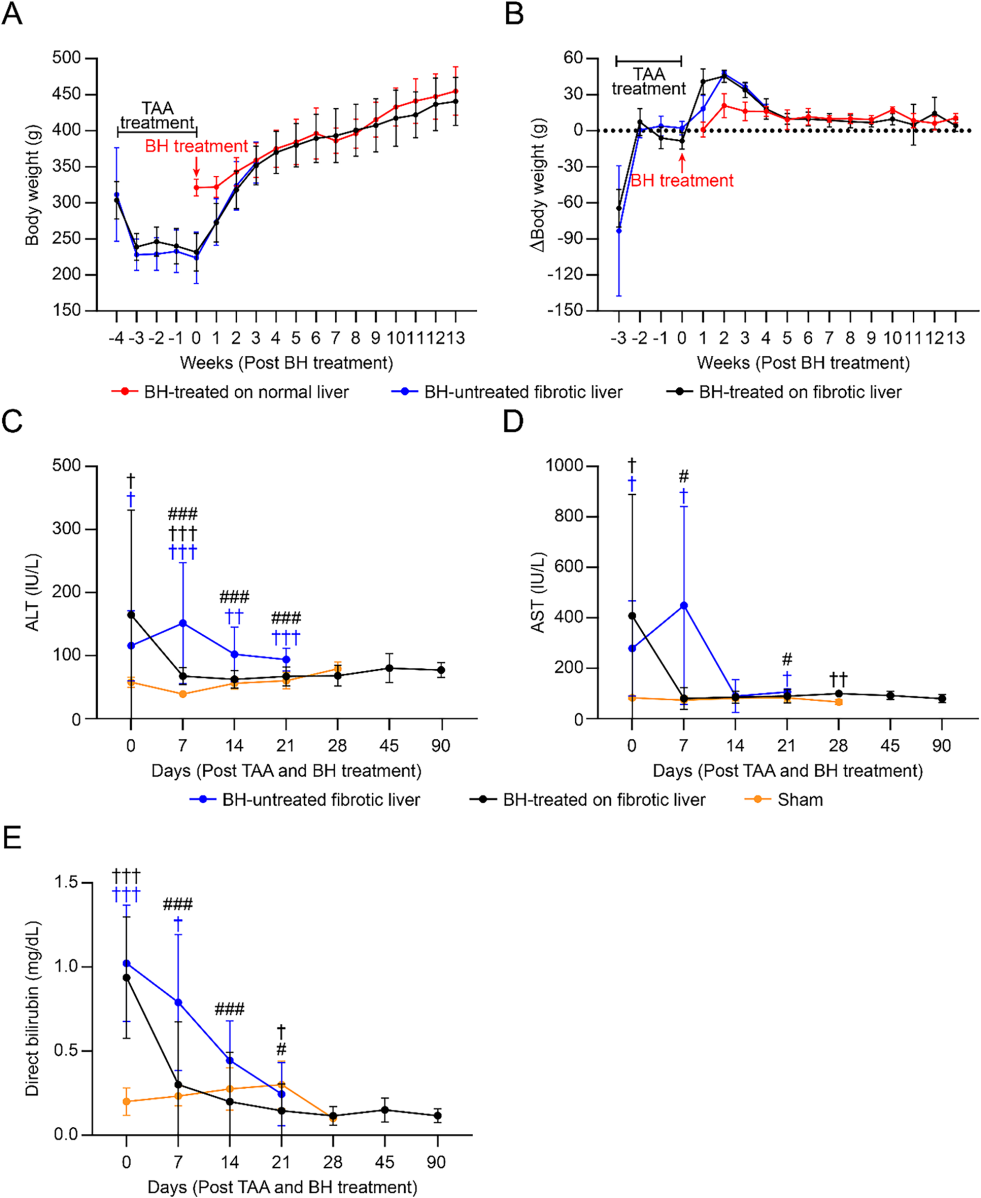
Recovery of surrogate markers of liver injury and body weight after TAA-induced liver fibrosis and BH treatment. (**A**) Changes in body weight after TAA-induced liver fibrosis and BH treatment. Week −4 to −1: n = 8 for BH-untreated fibrotic liver, n = 13 for BH-treated on fibrotic liver, Week 0 to 3: n = 9 for BH-treated on normal liver, n = 8 for BH-untreated fibrotic liver, n = 13 for BH-treated on fibrotic liver, Week 4 to 6: n = 9 for BH-treated on normal liver, n = 0 for BH-untreated fibrotic liver, n = 13 for BH-treated on fibrotic liver, Week 7 to 13: n = 9 for BH-treated on normal liver, n = 0 for BH-untreated fibrotic liver, n = 10 for BH-treated on fibrotic liver. Data are presented as means ± SD. One–way ANOVA followed by a post-hoc Tukey’s test was used. No significant change was observed. (**B**) Changes in body weight differences during a week (ΔBody weight: Week n + 1 body weight − Week n body weight) after TAA-induced liver fibrosis and BH treatment. (**C–E**) Changes in serum AST, ALT, and direct bilirubin in response to TAA-induced liver fibrosis and BH treatment. Day 0 to 14: n = 4 for Sham, n = 8 for BH–untreated fibrotic liver, n = 24 for BH-treated on fibrotic liver, Day 21: n = 4 for Sham, n = 8 for BH-untreated fibrotic liver, n = 22 for BH-treated on fibrotic liver, Day 21: n = 4 for Sham, n = 8 for BH-untreated fibrotic liver, n = 22 for BH-treated on fibrotic liver, Day 28: n = 4 for Sham, n = 0 for BH-untreated fibrotic liver, n = 13 for BH-treated on fibrotic liver, Day 45 to 90: n = 4 for Sham, n = 0 for BH-untreated fibrotic liver, n = 6 for BH-treated on fibrotic liver. All values are shown as means ± SD (^†^*P* < 0.05 vs. Sham, ^††^*P* < 0.01 vs. Sham, ^†††^*P* < 0.001 vs. Sham, ^#^*P* < 0.05 vs. BH-untreated fibrotic liver, ^###^*P* < 0.001 vs. BH-untreated fibrotic liver).

In comparison to the group with TAA-induced liver cirrhosis and the group treated with BH but without liver cirrhosis, BH treatment for TAA-induced cirrhotic livers did not result in a statistically significant delay in body weight recovery (Fig. 6A). In addition, there was no statistically significant difference observed in the daily body weight reduction or recovery rate among the three groups (Fig. 6B). As shown in Fig. 6C and D, the BH treatment significantly reduced the TAA-induced elevation in serum AST and ALT levels. Furthermore, it also reduced serum bilirubin levels, which were increased in animal models with liver fibrosis (Fig. 6E).

## Discussion

Uncontrolled liver fibrosis progresses to liver cirrhosis, which is a major contributor to liver failure and liver cancer^3,4^. Discovering approaches to attenuate liver fibrosis, thereby preventing the progression of liver cirrhosis or liver cancer, could have a significant clinical benefit. In this context, we have clearly demonstrated, for the first time, that BH treatment effectively reduces liver fibrosis and promotes liver regeneration in an animal model of liver fibrosis.

This study clearly reported the long-term feasibility of inducing localised therapeutic effects by BH. The anti-fibrotic effect was highlighted in the BH-treated penumbra and the BH-treated identical lobe from 21 to 90 days after BH treatment on the fibrotic liver. Moreover, liver regeneration was also facilitated in the BH-treated penumbra from 7 to 90 days and in the BH-treated identical lobe from 21 to 90 days after BH treatment, respectively. Based on our results, BH treatment seems to destroy fibrotic tissue, thereby stimulating the proliferation of hepatocytes in both the BH-treated penumbra and the BH-treated identical lobe, resulting in an anti-fibrotic effect.

In this experimental study, the BH-treated core exhibited a higher liver fibrosis score (Fig. 2) and α-SMA expression (Fig. 3A) compared to both the BH-treated and BH-untreated lobes in the acute phase (7 days after BH treatment). This increase gradually decreased through days 21 and 90 after BH treatment. In line with these findings, our recent study on normal liver tissue revealed that the BH-treated core exhibited higher levels of α-SMA expression and collagen deposition compared to both the BH-treated penumbra and the BH-untreated area. These elevated levels gradually returned to normal values over the course of days 14 to 28, reflecting the normal healing process^16^. Consequently, the heightened fibrosis observed in the BH-treated core during the acute phase following BH treatment in the TAA-induced liver fibrosis animal model could be attributed to the combined effects of the normal healing process.

Accumulating evidence suggests that activated myofibroblasts, primarily hepatic stellate cells with α-SMA (+) and producers of collagen type I, play a key role in the progression of liver fibrosis.^20,22,23^. Furthermore, the clearance of activated myofibroblasts is associated with the regression of liver fibrosis, leading to an emerging therapeutic target for novel treatment against liver fibrosis^20,22,23^. Indeed, the reduction of activated myofibroblast populations can attenuate the collagen fiber production, thereby reducing liver fibrosis. In this study, we found that BH treatment promoted the reduction of activated myofibroblasts in all BH-treated lobes compared to BH-untreated lobes at 21 and 90 days after BH treatment. Notably, α-SMA expression in BH-treated lobes was nearly returned to normal values 90 days after BH treatment.

In terms of safety, we observed that BH-treatment did not exacerbate the inflammatory response in the treated fibrotic liver compared to the sham animal and showed no adverse side effects on the treated animals. Consistent with our results, the First-in-Human study (NCT03741088) of histotripsy treatment on multifocal liver tumours also reports no adverse clinical side effects^24^. A histotripsy device (Edison®, HistoSonics, USA) to treat patients with liver tumours has recently been approved by the US Food and Drug Administration (FDA) in Oct 2023^25^.

Liver transplantation is regarded as a cure of both cancer and cirrhosis^26,27^. However, patients on the waiting list for liver transplantation are often delayed or delisted due to a shortage of available liver donors and deteriorated liver function beyond the accepted criteria for transplantation^26,27^. Thus, currently, to maximize the clinical benefit of liver transplantation, interventional therapy called Bridging therapy is widely performed to prevent patients from advancing beyond the specified criteria while awaiting an organ^27,28^. In this perspective, BH treatment could serve as a promising modality for Bridging Therapy, given its ability to inhibit liver fibrosis and promote liver regeneration.

Taken together, this study highlights the first in vivo investigation reporting the therapeutic efficacy of BH on animal models with fibrotic livers. BH treatment showed an excellent anti-fibrotic effect without any adverse side effects in fibrotic liver tissues. Therefore, it could be a promising novel therapeutic modality for the treatment of liver fibrosis in the future.

## Methods

### Induction of liver fibrosis

Sprague–Dawley rats (Male, 6 weeks old, body weight of 260 to 280 g) were from Koatech (Pyeongtaek, Republic of Korea) and kept under a 12-h light/dark cycle, with unrestricted access to water and food. Following a one-week period of adaptation, rats were subjected to intraperitoneal injections of either a vehicle solution (0.9% normal saline) or TAA (300 mg/kg, 12 mg/mL in normal saline) three times a week for 28 days. TAA is an organosulfur compound well-known for inducing hepatic fibrosis and is regarded as an ideal fibrosis-inducing agent to evaluate the anti-fibrotic effect in experimental animals^29^. The Institutional Animal Care and Use Committee (IACUC) at Korea University granted approval for all experimental procedures and protocols under the reference number KOREA-2022-0120. All the methods were performed in accordance with the relevant guidelines and regulations.

### Surgery for BH experiment

After 28 days of TAA administration, surgery for BH experiment was performed as previously mentioned^16^. Anaesthesia induction was performed by administering 3.5% isoflurane within a ventilated anaesthesia chamber, utilizing a mixture of nitrous oxide and oxygen. Maintenance of anaesthesia was achieved through administering 2 to 2.5% isoflurane through a nasal cone in a 2:1 mixture of nitrous oxide and oxygen via inhalation. Next, the rats were situated on a heated pad, and a midline xipho-pubic laparotomy was executed to unveil the LLL of the liver. After BH treatment, the incisional site was sutured, and the subjects were permitted unrestricted access to food and water upon awakening. The sham animals only underwent xipho-pubic laparotomy with wound closure, without TAA administration and BH treatment.

### BH experimental protocol

The same BH experimental protocol used in our previous study^16^ was adopted (Fig. 1A), which successfully demonstrated its efficacy and long-term safety on normal liver tissue in vivo^16^. A 2 MHz-HIFU transducer (H148, Sonic Concepts, Bothwell, WA, USA), which was triggered by a function generator (33600A, Keysight Technologies, Santa Rosa, CA, USA) in conjunction with a power amplifier (1040L, Electronics & Innovation, Rochester, NY, USA), was employed to generate a number of BH lesions in the fibrotic liver. A laser pointer, affixed to the translation stage and aligned with the central axis of the HIFU source, was used for the guidance of the BH treatment onto the target surface of the exposed LLL of the liver. During our experiments, the HIFU focus was kept constant as 5 mm below the surface of the liver. Ten 2 MHz HIFU pulses each comprising a 10 ms pulse duration, *P*_+_ of 89.1 MPa, *P*_−_ of −14.6 MPa with 1% DC and 1 Hz PRF were used to produce a single BH lesion at a given target position in the fibrotic liver. BH lesions were generated using a raster-scanning approach. The HIFU focus was systemically traversed across an area of 1.44 cm^2^ (1.2 cm × 1.2 cm) in both the transverse and lateral directions with 2 mm step increments.

### Histopathology

Rats were euthanised 7, 21, and 90 days after BH treatment by carbon dioxide inhalation. The regions to determine the fibrosis score for BH-treated groups on the liver tissue (LLL) were carefully selected as previously described^16^. The BH-treated core was chosen as the region treated with the BH. The BH-treated penumbra was selected from the same histological slides surrounding the BH-treated core. The top right part of the left lateral lobe (LLL) and the center of the median lobe (ML) were selected for the BH-untreated identical lobe (LLL) and BH-untreated (ML), respectively. Next, the liver tissues were fixed for 48 h in a 4% paraformaldehyde solution, followed by desiccation in 70% ethanol. Subsequently, the tissues were embedded in a paraffin block. Next, blocks were sectioned to a thickness of 4.5 μm and subsequently subjected to staining procedures utilising haematoxylin and eosin (H&E) as well as Masson's trichrome (MT) staining for histological examination. All stained images were assessed using the Zeiss Axio Scan Z1 (Carl Zeiss, Jena, Germany). The degree of fibrosis was also quantified using the Ishak scoring system^30^.

### Immunohistochemistry

Sectioned liver tissues were incubated at 4 °C with following primary antibodies: anti-ASGR1 (1:200 dilution, ab127896, Abcam, Cambridge, MA, USA), anti-CD68 (1:200 dilution, ab31630, Abcam, Cambridge, MA, USA), anti-CD26 (1:200 dilution, ab187048, Abcam, Cambridge, MA, USA), and anti-α-SMA (1:400 dilution, ab7817, Abcam, Cambridge, MA, USA). Next, Alexa Fluor 488-conjugated goat anti-rabbit IgG (A11034, Carlsbad, CA, USA) and Alexa Fluor 555-conjugated goat anti-mouse IgG (A21424, Carlsbad, CA, USA) were employed as the secondary antibodies. The cell nuclei were subsequently counterstained with 4′,6-diamidino-2-phenylindole (DAPI). All fluorescence images were obtained using a slide scanner (Zeiss Axio Scan Z1, Carl Zeiss, Jena, Germany). For scoring of ASGR1, CD26, and CD68, we measured the intensity of the fluorescence signal within the square-shaped ROIs (962.7 μm × 962.7 μm) using ImageJ software version 1.54d (NIH Image, Bethesda, MD, USA; https://imageJ.net). In the present study, we used ASGR1, which is widely accepted as a hepatocyte-specific marker in the field of hepatology^19^. Furthermore, ASGR1 can serve as a specific target molecule for the selective delivery of drugs or small molecules into hepatocytes^31^. In this context, we believe that ASGR1 is significant in representing the restoration of hepatocyte population in fibrotic liver.

### Quantitative real-time RT-PCR analysis (qRT-PCR)

Total RNA was isolated from liver tissue samples using Rneasy Fibrous Tissue Mini Kit (Qiagen, Hilden, Germany), and cDNA synthesis was conducted through the application of iScript cDNA Synthesis Kit (Bio-Rad, Hercules, CA, USA). Gene expression was assessed using iCycler PCR thermocycler (Bio-Rad, Hercules, CA, USA) and SYBR Green mixture (iQTM SYBR Green Supermix, Bio-Rad, Hercules, CA, USA). Specific mRNA template sets were created based on GenScript (Piscataway, NJ, USA). The mRNA levels were subsequently standardized using glyceraldehyde 3-phosphate dehydrogenase (GAPDH) as the reference^32^. The sequences of primer pairs for the genes of interest are as follows: Collagen type I alpha 1 chain (COL1A1) (5′-GATCTCCTGGTGCTGATGGA-3′ and 5′-GACCAGGGAAGCCTCTTTCT-3′), collagen type III alpha 1 chain (COL3A1) (5′-ACTGGTGAACGTGGCTCTAA-3′ and 5′-GGACCTGGATGTCCACTTGA-3′), GAPDH (5′-CAAGGCTGAGAATGGGAAGC-3′ and 5′-GAAGACGCCAGTAGACTCCA-3′), α-SMA (5′-GCTATTCAGGCTGTGCTGTC-3′ and 5′-GTTGTGAGTCACGCCATCTC-3′), and Vimentin (5′-ATGAAA GTGTGGCTGCCAAGAAC-3′ and 5′-GTGACTGCACCTGTCTCCGGTA-3′). The scoring of α-SMA, vimentin, and collagen was determined by the quantity of transcriptomes (mRNA) present within homogenized liver tissue.

### Hydroxyproline assay

The total collagen content in the liver was quantified using a colorimetric hydroxyproline assay kit (ab222941, Abcam, Cambridge, MA, USA). Liver tissues were denatured with a papain solution, as previously described^33^, and the hydroxyproline assay was performed according to the manufacturer’s instructions.

### Blood analysis

The quantification of serum levels of aspartate aminotransferase (AST), alanine aminotransferase (ALT), and direct bilirubin was performed utilizing the FUJI DRI-Chemiclinical Chemistry Analyzer (FUJI DRI-CHEM 4000i, Fuji Film, Tokyo, Japan).

### Statistical analysis

All data were expressed in terms of the mean and standard deviation. The normal distribution was assessed using the Shapiro–Wilk test. Parametric analysis employed a one-way analysis of variance (ANOVA) with subsequent post-hoc correction using Tukey’s test, whereas non-parametric analysis was conducted through the Kruskal–Wallis test, followed by post-hoc testing utilizing the Conover test. Data analysis was conducted using MedCalc software version 18.5 (MedCalc Software Ltd, Ostend, Belgium) and SPSS software version 17.0 (SPSS Inc, Chicago, IL, USA). A *P*-value less than 0.05 was considered statistically significant.

### Ethical approval

This study was reported in accordance with ARRIVE guidelines.

## Data Availability

The data that support the findings of this study are available from the corresponding author upon reasonable request.
